# Spatial inequality, characteristics of internal migration, and pulmonary tuberculosis in China, 2011–2017: a spatial analysis

**DOI:** 10.1186/s40249-020-00778-0

**Published:** 2020-11-19

**Authors:** Wen-Chong He, Ke Ju, Ya-Min Gao, Pei Zhang, Yin-Xia Zhang, Ye Jiang, Wei-Bin Liao

**Affiliations:** 1 Research Management Office, West China Second University Hospital, Sichuan University, Chengdu, China; 2 Key Laboratory of Birth Defects and Related Diseases of Women and Children (Sichuan University), Ministry of Education, Chengdu, China; 3 Department of Epidemiology and Preventive Medicine, School of Public Health and Preventive Medicine, Monash University, Melbourne, Australia; 4 West China School of Public Health and West China Fourth Hospital, Sichuan University, Chengdu, China; 5 Department of Health, Northwest Minzu University, Lanzhou, China; 6 School of Public Health, The University of Hong Kong, Hong Kong, Hong Kong Special Administrative Region China; 7 School of Geography and Environmental Engineering, Lanzhou City University, Lanzhou, China

**Keywords:** Internal migration, Pulmonary tuberculosis, Spatial analysis, China

## Abstract

**Background:**

Human migration facilitate the spread of tuberculosis (TB). Migrants face an increased risk of TB infection. In this study, we aim to explore the spatial inequity of sputum smear-positive pulmonary TB (SS + PTB) in China; and the spatial heterogeneity between SS + PTB and internal migration.

**Methods:**

Notified SS + PTB cases in 31 provinces in mainland China were obtained from the national web-based PTB surveillance system database. Internal migrant data were extracted from the report on China’s migrant population development. Spatial autocorrelations were explored using the global Moran’s statistic and local indicators of spatial association. The spatial variation in temporal trends was performed using Kulldorff’s scan statistic. Fixed effect and spatial autoregressive models were used to explore the spatial inequity between SS + PTB and internal migration.

**Results:**

A total of 2 380 233 SS + PTB cases were reported in China between 2011 and 2017, of which, 1 716 382 (72.11%) were male and 663 851 (27.89%) were female. Over 70% of internal migrants were from rural households and had lower income and less education. The spatial variation in temporal trend results showed that there was an 9.9% average annual decrease in the notification rate of SS + PTB from 2011 to 2017; and spatial clustering of SS + PTB cases was mainly located in western and southern China. The spatial autocorrelation results revealed spatial clustering of internal migration each year (2011–2017), and the clusters were stable within most provinces. Internal emigration, urban-to-rural migration and GDP per capita were significantly associated with SS + PTB, further, internal emigration could explain more variation in SS + PTB in the eastern region in mainland. However, internal immigration and rural-to-urban migration were not significantly associated with SS + PTB across China.

**Conclusions:**

Our study found the spatial inequity between SS + PTB and internal migration. Internal emigration, urban-to-rural migration and GDP per capita were statistically associated with SS + PTB; the negative association was identified between internal emigration, urban-to-rural migration and SS + PTB. Further, we found those migrants with lower income and less education, and most of them were from rural households. These findings can help stakeholders to implement effective PTB control strategies for areas at high risk of PTB and those with high rates of internal migration.

## Background

Tuberculosis (TB), an infectious disease caused by mycobacterium TB, remains a major public health issue worldwide [1, 2]. The World Health Organization (WHO) estimated that 10.4 million people developed TB and there were more than 1.7 million TB deaths worldwide in 2016 [3]. TB was the second leading cause of morbidity and mortality among the class A and B infectious diseases in China [4]. Over the past two decades, China has made great progress in controlling TB; the prevalence of smear-positive TB cases has declined by 65% with the implementation of a national-scale control programme to tackle TB problems [5]. Despite this progress, there is still nearly one million newly confirmed cases annually. Moreover, there is spatial inequality in the prevalence of TB between the east, central, and western regions of China [6].

Human migration involves the movement and change residence of a person or group; migration can occur across a country border or within a state. The migration flow in China over the past two decades has increased due to rapid economic development and unprecedented urbanization. Before the reform of the household register system (*hukou* system), people were required to stay at their *hukou* place [7]. There was limited access to social security, insurance, and medical care when people left their *hukou* place [8]. Since reform of the economy in the 1980s, China has relaxed its *hukou* restrictions and many migrants now leave their homeland to seek jobs. In 2010, there were an estimated 221 million internal migrants in China (16.5% of the total population based on the census data in 2010) while in 2016, there were an estimated 245 million internal migrants in China (17.72% of the total population based on the national population change survey). Compared with permanent migrants, internal migrants face an increased risk of TB infection, low income, and poor living and working conditions [9, 10]. Furthermore, migrants themselves can influence the epidemiology of TB, especially in the case of people who have latent TB infection before or during migration [11, 12].

As mentioned above, migration refers to a geographic move from one area to another. However, despite the remarkable economic growth and the reduction in poverty in China, spatial inequality has grown and there is an uneven distribution in poverty reduction within the country [13]. Moreover, the economy and poverty are push and pull factors for migration; the push is driven by poverty or environmental disasters and the pull is driven by higher incomes and better lifestyle opportunities [14, 15]. Therefore, TB epidemiology is influenced by a combination of geographic and social factors. Previous studies have used the geographical information system (GIS) and spatial statistics to explore the spatial characteristics of TB [16–19]. The findings have revealed significant cluster patterns at the province, prefecture, and regional level, indicating that TB epidemiology is not randomly distributed.

Few studies have simultaneously examined the spatial inequality of TB and the characteristics of TB and internal migration in China. In our previous research, we focused on the association between TB and internal migration in China [20]. However, detailed examination of the characteristics of internal migrants was hampered by limited data. In this study, the internal migration factors included emigration, immigration, rural-to-urban migration and urban-to-rural migration. Emigration was defined as when a person left their household registration place for more than one month. Immigration was defined as when a person was settled in their current residence area for more than one month. Rural-to-urban migration was defined as when a person migrated from rural household registration place to urban area for more than one month. Urban-to-rural migrated from urban household registration place to rural area for more than one month. We first performed a detailed analysis of sputum smear-positive pulmonary TB (SS + PTB) and internal migration. We also attempted to identify the spatial inequality of SS + PTB and internal migration. Finally, the fixed effect and spatial autoregressive models were used to evaluate the association between spatial inequality of SS + PTB and internal migration at the provincial level.

## Methodology

### Data collection

Data for notified SS + PTB cases in 31 provinces (excluding Hong Kong, Macau, and Taiwan) were obtained from the national, web-based Notifiable Infectious Diseases Reporting Information System (NIDRIS). This database includes sputum smear-positive, sputum smear-negative, sputum not done, and sputum culture-positive PTB data. Due to its high risk of transmission among the population, SS + PTB is of greatest concern; therefore, we focused on SS + PTB cases within China from 2011 to 2017. The classification of eastern areas, central areas, and western areas was based on the standard of the National Statistics Bureau.

The internal migration data were extracted from the 2011–2017 report on China’s migrant population development. The reports are based on the National Dynamic Monitoring Survey on Migrants, which were conducted by the National Health and Family Planning Commission of China. This national survey has been conducted every year since 2009. Its main purpose is to analyze the social integration and health care of internal migrants. The internal migrants are drawn using a stratified multistage and Probability Proportional to Size (PPS) sampling method. The survey covers 31 provinces in mainland China with migrants aged 15–59 years who do not have the *hukou* of the survey city and have been living in the survey city for more than one month. The survey data includes demographic characteristics, social economic factors, public health and medical service utilization, and family planning services.

In the current study, internal migration was defined as a move from one province to another province within mainland China. Internal migration was divided into emigration and immigration. We then calculated the proportion of emigrants (POE), immigrants (POI), rural-to-urban migrants (POR) and urban-to-rural migrants (POU) in the total population for each province. Other variables included gross domestic product (GDP) per capita (CNY 10 000; PCGDP), the proportion of people in the population with a college degree or higher (EDU), the number of hospital beds per thousand (BED), the ratio of males to females (MF), the urbanization rate (the proportion of urban population in the total population, and the total population include the household registered population and the migration population, UR), and population density (persons per square kilometres; PD). Detailed information for these variables is shown in Table 1.

**Table 1:** Specification of the variables

Variables	Description of observed variables	Data source	Period
SS + PTB	Sputum smear-positive pulmonary TB	National Notifiable Infectious Diseases Reporting Information System	2011–2017
POE	The proportion of emigration in total population	Report on China’s Migrant Population Development	
POI	The proportion of immigration in total population		
POR	The proportion of rural to urban migration in total population		
POU	The proportion of urban to rural migration in total population		
PCGDP	GDP per capita (10000RMB)	China Statistical Yearbook	
EDU	The proportion of people with a college degree or above in total population		
UR	The proportion of urban population in total population		
PD	Population density (persons per square kilometres)		
MF	The ratio of male to female		
BED	The number of hospital beds		

*TB* tuberculosis, *SS + PTB* sputum smear-positive pulmonary TB, *POE* proportion of emigrants, *POI* proportion of immigrants, *POR* proportion of rural-to-urban migrants, *POU* proportion of urban-to-rural migrants, *PCGDP* gross domestic product per capita, *EDU* college degree or higher, *MF* the ratio of male to female, *UR* urbanization rate, *PD* population density, *BED* the number of hospital beds

### Data analysis

The statistical analysis for SS + PTB and internal migration data in three steps. First, the demographic characteristics of SS + PTB cases and internal migrants were presented. Then, the Spatial autocorrelation analysis and Spatial variation in temporal trends were used to identified risk areas of SS + PTB. In the end, the fixed effect and spatial autoregressive models were used to estimate the effects of internal migration, demographic factors, and socio-economic factors on SS + PTB incidence.

### Spatial autocorrelation analysis

Global Moran’s *I* statistic was used to measure spatial autocorrelation [21]. The value of Moran’s *I* usually ranges from −1 to 1, with positive values indicating a positive association and negative values indicating a negative association. A higher value approaching −1 or 1 indicates a stronger association. A value of 0 suggests a random distribution. The *Z*-statistic and *P*-value were used to evaluate the significance of Moran’s *I.* In the current study, a first-order queen continuity weights matrix was used to characterise the spatial relationships among the provinces in mainland China. Then, we calculated global Moran’s *I* statistic in GeoDa (version 1.6.7; GeoDa Center for Geospatial Analysis and Computation, Arizona State University, AZ, USA) in order to examine the spatial autocorrelations between SS + PTB, POE, and POI every year from 2011 to 2017 in the study area.

Local Moran’s *I* is a local indicator of spatial autocorrelation (LISA) [22]. As above, the value of local Moran’s *I* ranges from −1 to 1, with a positive value indicating clustering of similar values and a negative value indicating the opposite. The sum of local Moran’s *I* is proportional to global Moran’s *I*. In our study, LISA was used to describe the local spatial autocorrelation of POE and POI by calculating the local Moran’s *I*. The cluster maps were created in ArcGIS (version 10.5; ESRI Inc, Redlands, CA, USA).

### Panel data analysis

The data of notified SS + PTB cases and internal migration from 31 provinces in mainland China from 2011 to 2017 were strongly balanced panel data. Furthermore, the Direct Observed Treatment Short Course (DOTS) strategy was implemented in 31 provinces of China in 2009. The effects of unobserved heterogeneity, such as DOTS strategy, can be assumed as fixed parameters in the fixed effects model [23]. Therefore, the fixed-effect and spatial autoregressive models were used to estimate the effects of internal migration, demographic factors, and socio-economic factors on SS + PTB incidence.

GDP per capita (CNY 10 000), population density, education level, the ratio of males to females, and urbanization level were used to reflect the social-economic situation. GDP represents the level of economic development of a region. The number of hospital beds reflects the availability of healthcare resources. Education and urbanization levels can indirectly affect SS + PTB incidence via the effects of income or health education on TB prevention [24, 25]. The natural logarithm of each variable was used in the construction of the model. The model was expressed as:

1 $$Y_{i,t} = \alpha + POE_{i,t} \beta_{1} + POI_{i,t} \beta_{2} + X_{i,t} \beta + year + \varepsilon_{i,t}$$

where $Y_{i,t}$ is the incidence of SS + PTB, *i* and *t* are the province and year, respectively, $\alpha$ is the intercept term, $X_{i,t}$ is a vector of independent variables, $\beta_{{1}}$ and $\beta_{{2}}$ are the coefficients of internal migration, $\beta_{{}}$ is the coefficient of other variables, $year$ reflects the effect of temporal variables, and $\varepsilon_{i,t}$ is the error term. The descriptive analysis and the fixed effects model were performed in Stata (version 15.0; StataCorp, TX, USA).

### Spatial variation in temporal trends

The spatial variation in temporal trends was used to identify the areas with exceptionally different temporal trends [26, 27]. This method, based on Kulldorff’s scan statistic, assumes that the risk of TB within the scanning window is the same as that outside the window. A circular window is imposed on each location in turn; then, a number of circular windows that are flexible in both size and location are constructed. For each window, a likelihood is calculated, and the most likely cluster is defined as the window with the maximum likelihood; that is, the cluster least likely to be due to chance. Under the null hypothesis, the *P*-value is obtained from Monte Carlo hypothesis testing. In this study, the Poisson probability model was used, in which the number of cases in each location was under a Poisson distribution. The maximum number of replications for the Monte Carlo simulation was set to 999 and *P* < 0.05 was considered to be statistically significant.

**Fig. 1:**
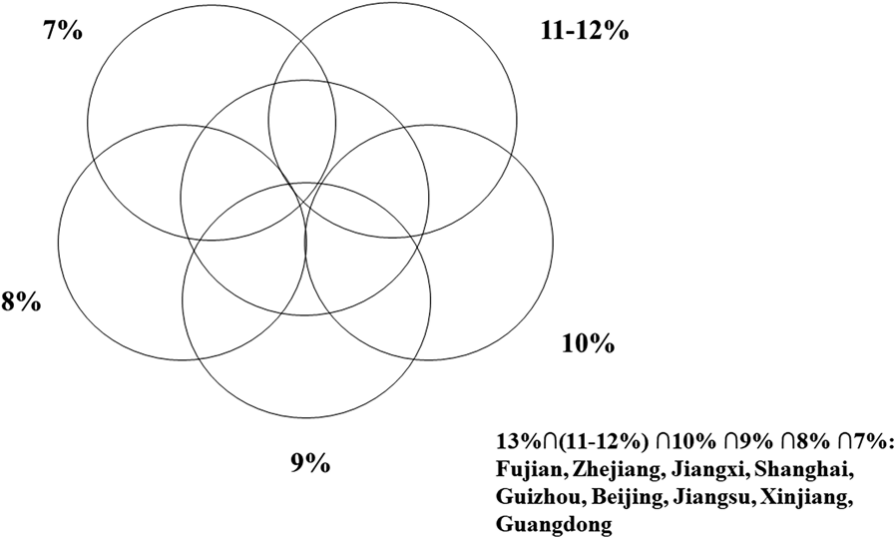
Venn diagram of spatial–temporal statistics for SS + PTB in mainland China, 2011–2017. SS + PTB: Sputum smear-positive pulmonary tuberculosis

Many studies using spatial-temporal statistics suggest that the results of spatial-temporal statistics are sensitive to the spatial and temporal scanning window [28, 29]. Further, some studies have suggested that the main criteria for selecting the optimal window are minimal overlapping areas and a single cluster making up no more than 15% of the whole study area [30, 31]. Moreover, previous research in China at the prefecture level reported that 11% of the total population at risk was the optimal spatial cluster size [32]. Therefore, we analysed the notified SS + PTB cases with a range of maximum spatial cluster sizes, from 5 to 20% of the total population at risk, at increments of 1%. The results showed that when the maximum spatial cluster size was between 7 and 13%, there was the least amount of overlap and the biggest cluster covered no more than 15% of all cities. As the impact of internal migration and the population at risk were dynamic, we chose the intersection result of 7% to 13% as the maximum spatial cluster size (see Fig. 1, Venn diagram of spatial-temporal statistics for SS + PTB in mainland China). The spatial–temporal clustering of notified SS + PTB cases was examined using SaTScan (version 9.6.0; Kulldorff and Information Management Services, Inc., Boston, USA).

## Results

### Descriptive analysis of SS + PTB cases

A total of 2 380 233 SS + PTB cases were reported in China between 2011 and 2017, of which, 1 716 382 (72.11%) were male and 663 851 (27.89%) were female. The notification rate of SS + PTB decreased from 29.82 cases per 100 000 population in 2011 to 16.78 cases per 100 000 population in 2017, with an annual average rate of 21.42 per 100 000 population. Table 2 shows that the number of male cases was twice that of female cases. In addition, a significant proportion of the SS + PTB infections were aged > 60 years old (33.92%) and between 45 and 60 years old (27.35%). Among the reported cases, around two-thirds were peasants; the percentage of SS + PTB cases that were classified as retired or unemployed increased over the years of the study.

**Table 2:** The demographic characteristics of SS + PTB cases in China from 2011 to 2017

	2011	2012	2013	2014	2015	2016	2017
*Gender*							
Male	28 9079 (72.30)	500 574 (72.23)	221 248 (72.09)	194 846 (72.14)	175 772 (71.94)	168 145 (71.72)	166 718 (71.98)
Female	110 744 (27.70)	192 412 (27.77)	85 662 (27.91)	75 255 (27.86)	68 571 (28.06)	66 312 (28.28)	64 895 (28.02)
*Age*							
0–15 year	1604 (0.47)	1309 (0.38)	1059 (0.35)	933 (0.35)	893 (0.37)	984 (0.42)	1055 (0.45)
15–30 year	92 617 (27.25)	74 868 (21.61)	65 097 (21.21)	54 807 (20.29)	47 009 (19.24)	44 992 (19.19)	41 890 (18.09)
30–45 year	87 885 (25.86)	72 674 (20.97)	62 546 (20.38)	52 179 (19.32)	44 312 (18.14)	41 311 (17.62)	38 909 (16.80)
45–60 year	104 238 (30.67)	91 130 (26.30)	81 904 (26.69)	73 211 (27.11)	66 616 (27.26)	62 986 (26.86)	76 209 (26.55)
> 60 year	113 479 (33.39)	106 512 (30.74)	96 304 (31.38)	88 971 (32.94)	85 513 (35.00)	84 184 (35.90)	88 273 (38.11)
*Occupation*							
Peasants	268 045 (67.04)	230 940 (66.65)	203 042 (66.16)	179 052 (66.29)	158 472 (64.86)	150 085 (64.86)	147 627 (63.74)
Workers	21 020 (5.26)	16 859 (4.87)	13 959 (4.55)	10 363 (3.84)	9616 (3.94)	9141 (3.94)	8538 (3.68)
Domestic unemployed	30 734 (7.69)	29 249 (8.44)	32 112 (10.46)	34 001 (12.59)	32 508 (13.30)	32 493 (13.30)	32 981 (14.24)
Students	11 561 (2.89)	9066 (2.62)	7673 (2.50)	6627 (2.45)	5861 (2.40)	6228 (2.40)	6925 (2.99)
Migrant workers	12 206 (3.05)	8912 (2.57)	6209 (2.02)	3844 (1.42)	3447 (1.41)	3209 (1.41)	2618 (1.13)
Retirees	13 707 (3.43)	13 039 (3.76)	13 483 (4.39)	13 560 (5.02)	13 811 (5.65)	13 876 (5.65)	14 282 (6.17)
Others	42 550 (10.4)	38 428 (11.09)	30 432 (9.92)	22 654 (8.39)	20 628 (8.44)	19 425 (8.44)	18 642 (8.05)

*SS + PTB* sputum smear-positive pulmonary tuberculosis

Table 3 shows the characteristics of internal migrants from 2011 to 2017. The sample consisted of 1 231 277 internal migrants, 53.62% of whom were male and 46.38% were female. Further, 78.52% of migrants were married, 84.53% had at least a middle school education, 76.11% had a monthly household per capita income of less than CNY 7000 (around USD 1000), and 61.14% only had rural medical insurance. In addition, a significant proportion of the migrants were from rural households (84.53%). Among the total sample of internal migrants, 51.19% of whom were migrated across provinces and 30.73% migrated across municipal jurisdictions within a province. Over 85% of internal migrants had left their place of household registration for work or business purposes. Other reasons for migration included study and training, which only accounted for around 15% of internal migrants.

**Table 3:** The demographic characteristics (percentage) of internal migrants in China from 2011 to 2017

Characteristics	2011	2012	2013	2014	2015	2016	2017
*Gender*							
Male	53.16	53.09	53.69	58.55	53.06	52.12	51.69
Female	46.84	46.91	46.31	41.45	46.94	47.88	48.31
*Marital status*							
Married	77.49	76.21	76.43	76.11	78.87	80.46	84.05
Otherwise	22.51	23.79	23.57	23.89	21.13	19.54	15.95
*Educational attainment*							
Primary school or below	16.5	16.08	14.87	13.89	15.21	14.7	17.04
Middle school	55.02	53.39	54.19	52.7	50.49	47.01	43.66
High school	15.09	15.18	15.42	20.55	21.74	22.3	21.9
College degree or above	13.39	15.37	15.53	12.85	12.56	15.99	17.4
*Monthly income, RMB*							
< 3000	36.43	26.12	19.51	15.3	10.64	8.8	15.16
3000–5000	37.8	36.53	35.09	33.39	30.42	26.79	30.26
5000–7000	16.07	21.78	25.89	27.44	29.01	29.14	21.2
> 7000	9.71	15.57	19.51	23.87	29.93	35.27	33.38
*Medical insurance*							
Urban basic health insurance	8.07	10.76	19.89	14.19	27.14	24.89	11.67
New rural cooperative medical insurance	54.75	60.48	60.09	60.1	66.12	63.18	63.29
Othetwise	37.18	28.76	20.02	15.05	6.74	11.93	25.04
*Types of migration*							
Between provinces	50.62	56.46	52.08	50.96	49.88	49.07	49.29
Between municipal jurisdictions within province	31.22	27.91	28.78	30.33	30.34	33.58	32.95
Within municipal jurisdiction	18.16	15.64	19.14	18.71	19.76	17.35	17.76
*Type of household*							
Rural	84.84	84.29	85.34	84.14	83.59	82.16	77.98
Other	15.16	15.71	14.66	15.86	16.41	17.84	22.02
*Reason of migration*							
Working or doing business	None	None	88.54	88.13	84.39	83.6	83.61
Others	None	None	11.46	11.87	15.61	16.4	16.39
*N*	128 000	158 556	198 795	200 937	206 000	169 000	169 989

**Fig. 2:**
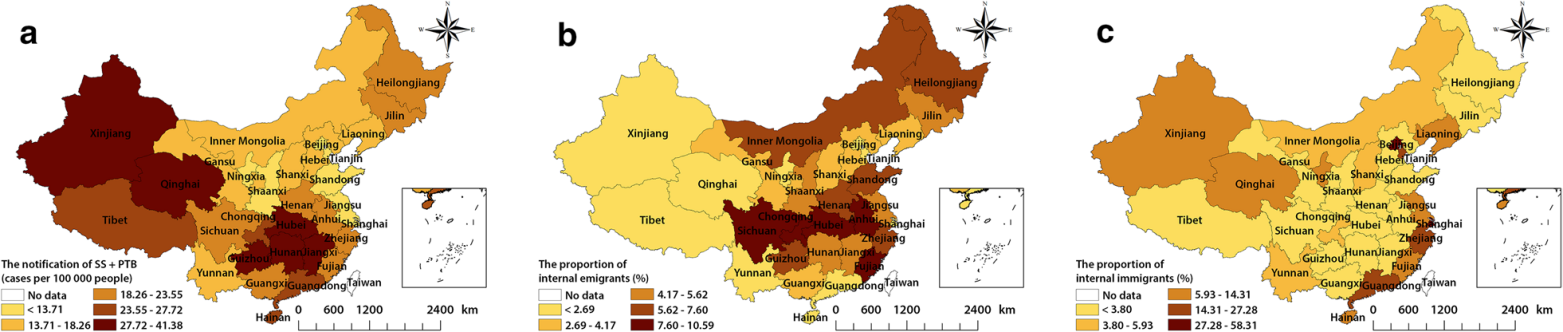
The annual average notification rate of SS + PTB and the proportions of emigrants/immigrants at the province level in mainland China, 2011–2017. **a** Illustrates the notification rate of SS + PTB. **b** and **c** illustrate the proportions of emigrants and immigrants, respectively. *TB* tuberculosis; *SS + PTB* sputum smear-positive pulmonary TB

Figure 2 shows the spatial distribution of the annual average notification rate of SS + PTB and the proportions of internal emigrants and immigrants in China at the provincial level from 2011 to 2017. There were obvious spatial variations in the annual average notification rate of SS + PTB, with rates ranging from 8.37 to 41.38 per 100 000 population. The highest SS + PTB notification rates were found in Xinjiang, Qinghai, Hubei, Hunan, Jiangxi, and Guizhou provinces, primarily in the northwest and south of China. In addition, the annual average notification rate of SS + PTB in 2011 to 2013 and 2014 to 2017 showed a similar spatial pattern. However, there was a significant decrease in the annual average notification rate of SS + PTB from 2014 to 2017, where great decrease of SS + PTB were found in Qinghai, Shanxi, Hainan, Gansu and Inner Mongolia provinces/municipalities (see Additional file 1: Figure S1).

Sichuan (10.59%), Fujian (9.81%), Anhui (9.19%), and Hubei (9.17%) provinces had the highest levels of internal emigrants. On the other hand, provinces with the highest levels of internal immigrants were located in eastern regions, such as Shanghai (58.31%), Beijing (51.99%), Tianjin (27.28%), Zhejiang (25.46%), and Guangdong (22.02%) provinces. Provinces with lower levels of immigrants were also located in southern areas close to Guangdong, Zhejiang, and Shanghai. However, those provinces had higher levels of internal emigrants.

### Global and local spatial autocorrelations

The global Moran’s *I* statistics showed positive spatial autocorrelations in SS + PTB in China each year (as presented in Table 4). Further, there was an increasing trend in global Moran’s *I* and *Z*-scores. The highest spatial autocorrelations were observed in 2013–2017, ranging from 0.384 to 0.413. Furthermore, the proportions of internal emigrants and immigrants were also spatially auto-correlated each year (see Table 5).

**Table 4:** Globe Moran’s *I* statistics of SS + PTB in China, 2011–2017

Year	Moran's *I*	*Z*-score	*P*-value	Pattern
2011	0.319	3.114	< 0.05	Clustered
2012	0.335	3.126	< 0.05	Clustered
2013	0.388	3.666	< 0.05	Clustered
2014	0.387	3.519	< 0.05	Clustered
2015	0.384	3.645	< 0.05	Clustered
2016	0.413	3.818	< 0.05	Clustered
2017	0.382	3.576	< 0.05	Clustered

*SS + PTB* sputum smear-positive pulmonary tuberculosis

**Table 5:** Globe Moran’s *I* statistics of emigrant and immigrant in China, 2011–2017

Year	Variable	Moran's *I*	*Z*-score	*P*-value	Pattern
2011	Emigrant	0.184	1.839	0.066	Not-clustered
2012	Emigrant	0.169	1.723	0.085	Not-clustered
2013	Emigrant	0.256	2.434	0.015	Clustered
2014	Emigrant	0.217	2.109	0.035	Clustered
2015	Emigrant	0.323	2.993	0.003	Clustered
2016	Emigrant	0.263	2.494	0.012	Clustered
2017	Emigrant	0.231	2.231	0.026	Clustered
2011	Immigrant	0.256	2.774	0.005	Clustered
2012	Immigrant	0.221	2.432	0.015	Clustered
2013	Immigrant	0.266	2.825	0.005	Clustered
2014	Immigrant	0.266	2.859	0.004	Clustered
2015	Immigrant	0.337	3.395	0.001	Clustered
2016	Immigrant	0.299	3.114	0.002	Clustered
2017	Immigrant	0.31	3.178	0.001	Clustered

**Fig. 3:**
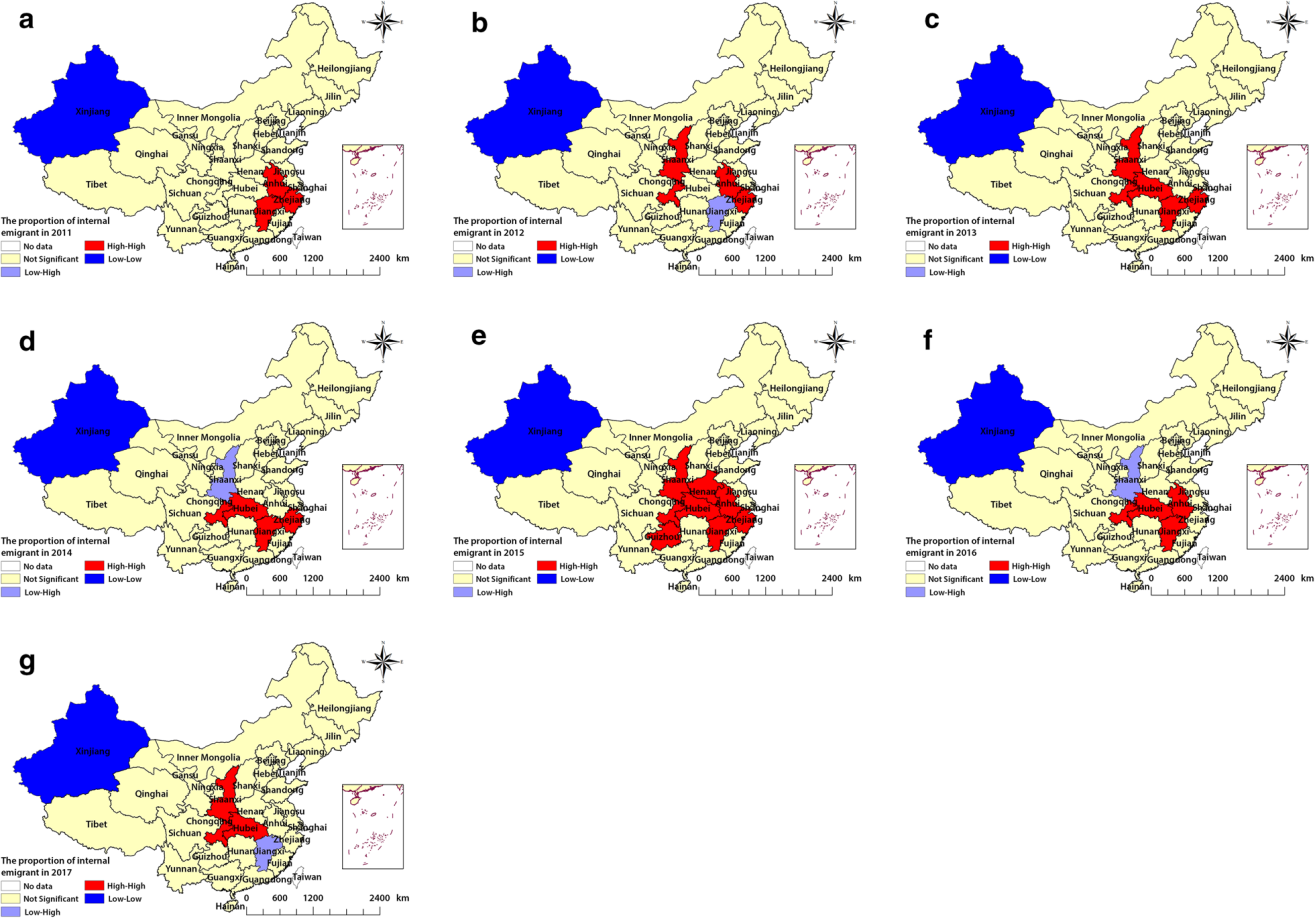
The LISA cluster map of internal emigrants in China. **a**, **b**, **c**, **d**, **e**, **f** and **g** show the spatial clustering of emigrants in 2011–2017, respectively. *LISA* local indicator of spatial association

**Fig. 4:**
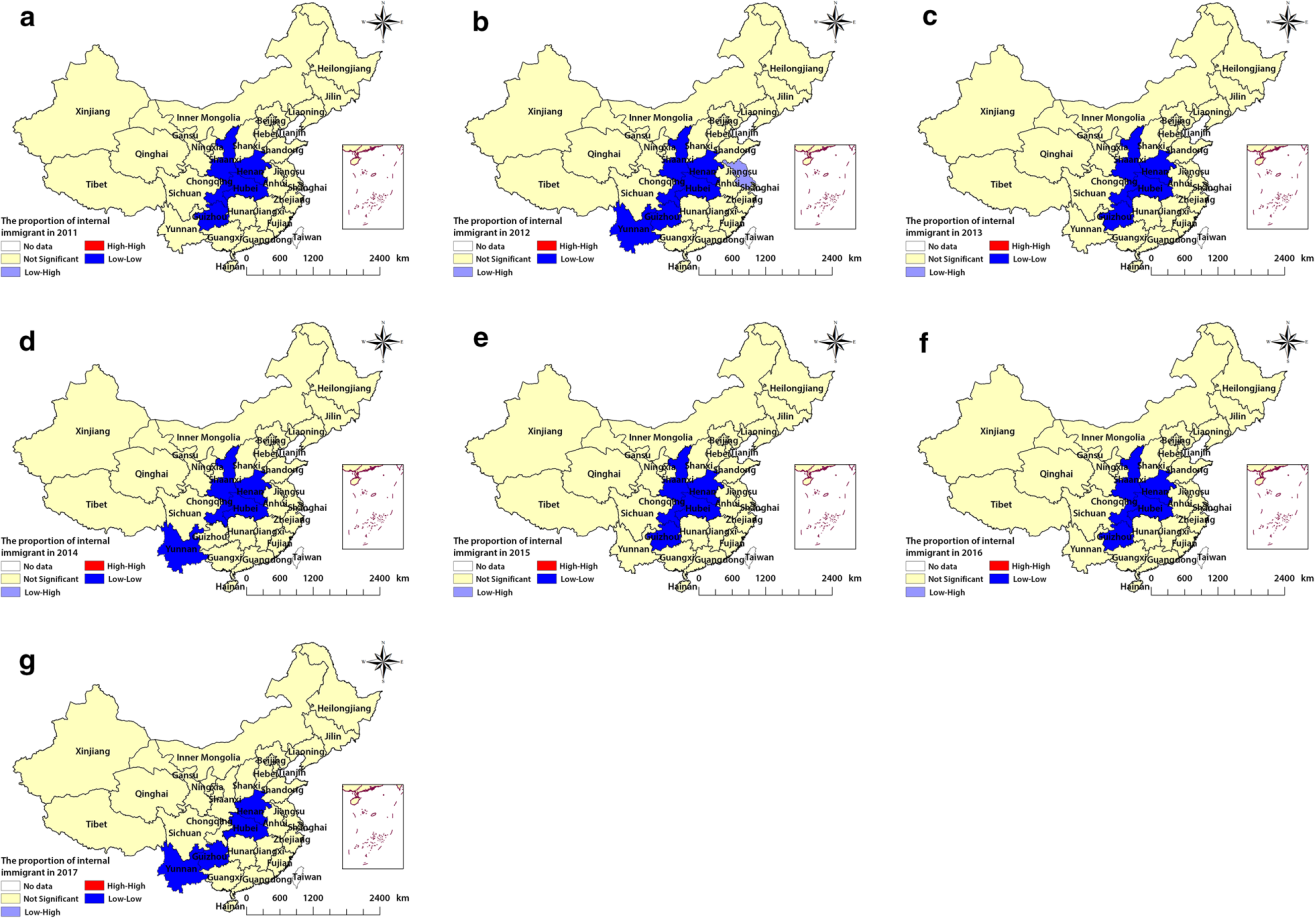
The LISA cluster map of internal immigrants in China. **a**, **b**, **c**, **d**, **e**, **f** and **g** show the spatial clustering of immigrants in 2011–2017, respectively. *LISA* local indicator of spatial association

Figures 3 and 4 show the local Moran’s *I* statistic results. Stability of spatial clusters was observed each year during the study period, and the clusters were stable within most provinces. Provinces such as Shaanxi, Henan, Chongqing, Guizhou, and Hubei showed a low-low type of relationship, indicating that these provinces had a low proportion of internal immigrants and the surrounding provinces also had low proportions of immigrants. Jiangsu Province, which is located on the southeast coast of China, had a low–high type of relationship, meaning that a low proportion of immigrants were found in Jiangsu while the surrounding provinces had high proportions of immigrants. Anhui, Jiangxi, Chongqing, Shaanxi, Guizhou, Henan, Hubei, and Zhejiang exhibited high-high types of relationships in the proportion of internal emigrants.

### The association between internal migration and SS + PTB

The fixed-effect and spatial autoregressive models were examined: one was the fixed-effect model (model 1 and 4), one was the spatial autoregressive model with continuity weights matrix (model 2 and 5), one was the spatial autoregressive model with distance decay weights matrix (model 3 and 6). The panel regression results indicated that POE, GDP per capita, population density, education level, and the ratio of males to females were significantly associated with the incidence of SS + PTB (see Table 6). Furthermore, population density and GDP per capita were significantly positively related to SS + PTB while the POE, education level and the ratio of males to females were significantly negatively related to SS + PTB. While POR was not significantly related to SS + PTB in model 4–6, POU was significantly negatively related to SS + PTB in model 6, and neither POR nor POU were significantly associated with the SS + PTB in model 4 and 5 (see Table 7). Moreover, neither POE nor POI were significantly associated with the SS + PTB in model S1-S3, and model S1 had the highest R-square value (see Additional file 1: Table S1).

**Table 6:** Spatial clusters of temporal trends of smear positive PTB in China, 2011–2017

Cluster	Province	Observed cases	Expected cases	Inside time trend	Out time trend	*RR*	*LLR*	*P-*value
Most likely cluster	Fujian, Zhejiang, Jiangxi, Shanghai	273 797	242 928	−3.85	−10.82	1.15	2656.66	< 0.001
Secondary cluster 1	Guizhou	84 996	52 590	−3.87	−10.15	1.64	739.22	< 0.001
Secondary cluster 2	Beijing, Tianjin	24 959	53 147	−2.69	−9.96	0.46	294.26	< 0.001
Secondary cluster 3	Jiangsu	74 598	118 993	−5.89	−10.06	0.61	290.55	< 0.001
Secondary cluster 4	Xinjiang	70 733	37 075	−5.79	−10.03	1.94	281.90	< 0.001
Secondary cluster 5	Ningxia	6108	9814	+ 0.94	−9.93	0.62	157.48	< 0.001
Secondary cluster 6	Tibet	6038	4720	−3.19	−9.92	1.28	62.44	< 0.001
Secondary cluster 7	Hainan, Guangxi	83 704	84 273	−9.33	−9.92	0.99	6.86	< 0.01

‘ + ’ means annual increase trend, ‘−’ means annual decrease trend; *PTB* pulmonary tuberculosis

**Table 7:** The result of fixed effect and spatial autoregressive model

Variable	Model 1	Model 2	Model 3
lnPOE	−0.103 (0.049)**	−0.101 (0.046)**	−0.104 (0.046)**
lnPOI	0.105 (0.065)	0.103 (0.055)*	0.105 (0.055)*
lnPCGDP	0.964 (0.369)**	0.855 (0.197)***	0.995 (0.185)***
lnPD	2.273 (1.488)	2.146 (0.716)**	2.152 (0.715)**
lnEDU	−0.152 (0.077)*	0.145 (0.074)**	−0.145 (0.074)**
lnUR	0.649 (0.595)	−0.607 (0.363)*	−0.627 (0.362)*
lnBED	−0.158 (0.209)	−0.142 (0.138)	−0.159 (0.138)
lnMF	−0.725 (0.517)	−0.771 (0.37)**	−0.69 (0.369)*
Year			
2012	−0.235 (0.053)***	−0.202 (0.045)***	−0.308 (0.062)***
2013	−0.439 (0.082)***	−0.376 (0.063)***	−0.568 (0.098)***
2014	−0.651 (0.117)***	−0.558 (0.083)***	−0.849 (0.142)***
2015	−0.772 (0.147)***	−0.659 (0.01)***	−1.019 (0.176)***
2016	−0.854 (0.168)***	−0.73 (0.11)***	−1.119 (0.19)***
2017	−0.895 (0.194)***	−0.768 (0.12)***	−1.159 (0.194)***
Intercept	−3.522 (6.841)		
ρ		−0.475 (0.292)	−0.441 (0.289)
No. Obs	217	217	217
R-squared	0.148	0.146	0.146

Robust stand-errors are in parentheses. ***, ** and *indicate the significance at 1%, 5%, and 10% level, respectively. *POE* proportion of internal emigrants (%), *POI* proportion of internal immigrants (%), *PCGDP* per capita GDP (10 000 RMB), *PD* population density (1/km^2^), *EDU* proportion of population with college degree or above (%), *UR* urbanization rate (%), *BED* the number of hospital beds, *MF* the ratio of male to female

### Spatial variation in temporal trends

**Fig. 5:**
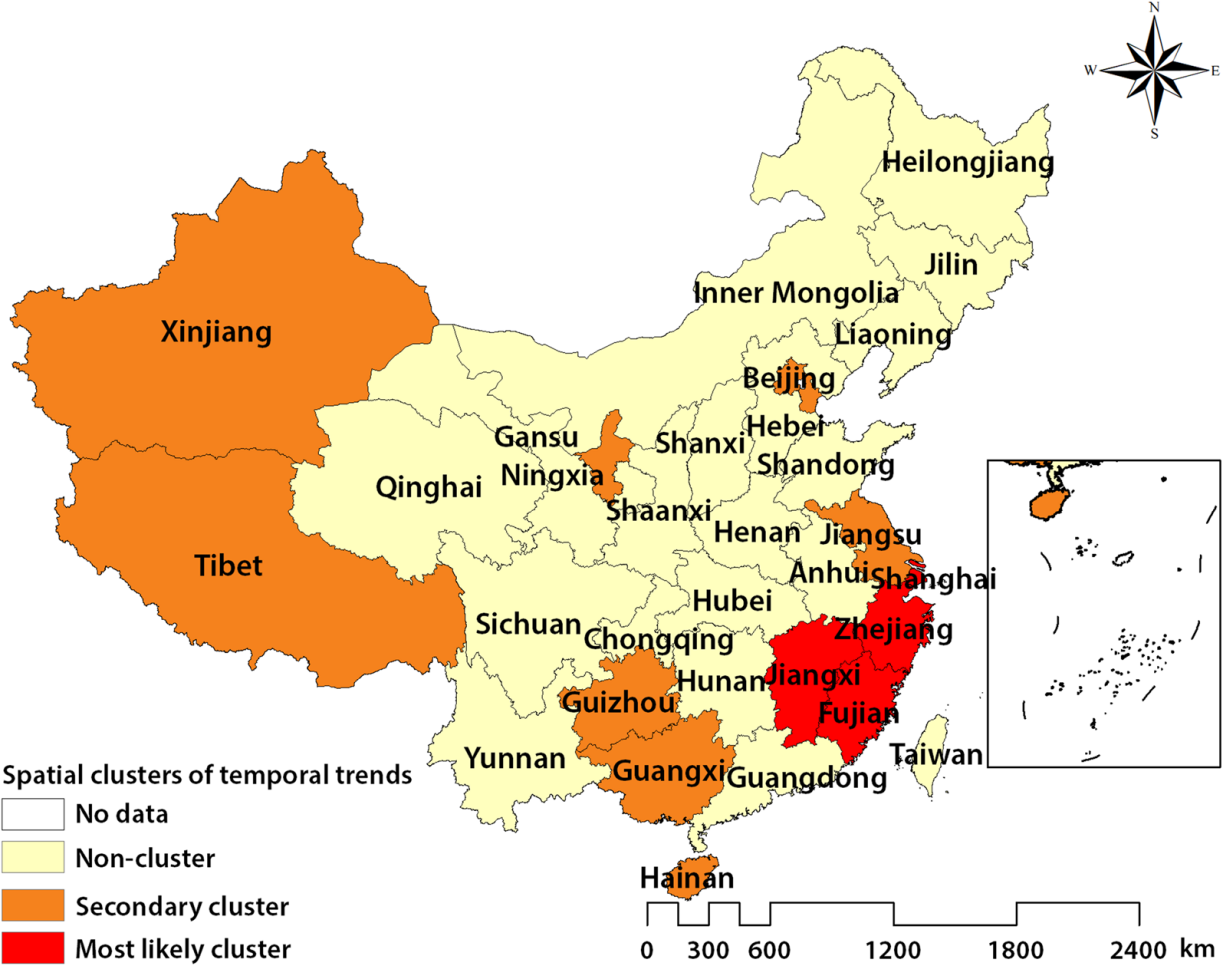
The spatial variation in temporal trends of smear-positive PTB in China, 2011–2017. *PTB* pulmonary tuberculosis

The spatial variation in temporal trend results showed that there was an 9.9% average annual decrease in the notification rate of SS + PTB from 2011 to 2017. One most likely cluster and seven secondary clusters were identified during the study period; one municipality showed increasing annual trends while 12 provinces/municipalities showed slower decreasing annual trends compared to the outside time trend (see Table 8). Ningxia showed an increasing annual average trend of 0.937%. Fujian, Zhejiang, Jiangxi, and Shanghai showed decreasing annual average trends of 3.846% compared to the outside time trend (10.818% annual decrease). Guizhou, Beijing, Tianjing, Jiangsu, Xinjiang, Tibet, Hainan and Guangxi showed decreasing annual average trends of 3.869%, 2.692%, 5.890%, 5.787%, 3.188%, and 9.327%, respectively. Figure 5 shows the spatial distribution of the most likely and secondary clusters. Most clusters were located in the southern provinces of China; although, Xinjiang, Ningxia, and Tibet are in west China and Beijing is in northeast China.

**Table 8:** The result of fixed effect and spatial autoregressive model

Variable	Model 4	Model 5	Model 6
lnPOR	0.085 (0.051)	0.076 (0.06)	0.089 (0.059)
lnPOU	−0.047 (0.065)	−0.041 (0.026)	−0.051 (0.026)**
lnPCGDP	1.01 (0.389)**	0.92 (0.197)***	1.042 (0.184)***
lnPD	2.153 (1.485)	2.04 (0.72)**	2.024 (0.715)**
lnEDU	−0.17 (0.078)**	−0.163 (0.075)**	−0.163 (0.075)**
lnUR	−0.595 (0.578)	−0.565 (0.366)	−0.567 (0.363)
lnBED	−0.199 (0.226)	−0.187 (0.139)	−0.201 (0.138)
lnMF	−0.722 (0.528)	−0.759 (0.372)**	−0.687 (0.37)*
Year			
2012	−0.28 (0.054)***	−0.248 (0.049)***	−0.361 (0.065)***
2013	−0.477 (0.084)***	−0.42 (0.067)***	−0.619 (0.101)***
2014	−0.684 (0.118)***	−0.602 (0.087)***	−0.898 (0.144)***
2015	−0.808 (0.148)***	−0.348 (0.104)***	−1.076 (0.179)***
2016	−0.88 (0.169)***	−0.773 (0.113)***	−1.166 (0.192)***
2017	−0.91 (0.195)***	−0.802 (0.122)***	−1.194 (0.196)***
Intercept	−3.174 (6.93)		
ρ		0.12 (0.093)	−0.475 (0.292)
No. Obs	217	217	217
R-squared	0.148	0.139	0.137

Robust stand-errors are in parentheses. ***, ** and *indicate the significance at 1%, 5%, and 10% level, respectively. *POR* proportion of rural-to-urban migrants (%); *POU* proportion of urban-to-rural migrants (%); *PCGDP* Per capita GDP (10 000 RMB); *PD* population density (1/km^2^); *EDU* proportion of population with college degree or above (%); *UR* urbanization rate (%); *BED* the number of hospital beds; *MF* the ratio of male to female

The 12.86% and 5.64% average annual decrease were found in the notification rate of SS + PTB from 2011 to 2013, 2014 to 2017. Beijing, Guizhou, Chongqing, Yunnan, Guangxi and Shaanxi showed an increasing annual average trend of 5.001%, 2.137% and 11.396% (see Additional file 1: Tables S2 and S3). Eight provinces/municipalities were located in western China; 5 provinces/municipalities were located in eastern China (see Additional file 1: Figure S2).

### Internal migration flow maps

Based on the SS + PTB spatial cluster results and panel data analysis, the most likely cluster and the six secondary clusters were chosen to produce internal migration flow maps. Among these clusters, Guangdong, Beijing, Shanghai, Fujian, Jiangsu, and Zhejiang are developed and prosperous provinces, while Guizhou and Jiangxi are located in southern China, near Guangdong, Fujian, and Zhejiang provinces, which have large immigrant populations. The proportion of emigrants was significantly higher than the proportion of immigrants in Guizhou (POE: 6.32% vs POI: 3.09%) and Jiangxi (POE: 5.08% vs POI: 1.42%). In contrast, the proportion of immigrants was obviously higher than the proportions of emigrants in Guangdong (POI: 22.02% vs POE: 2.59%), Beijing (POI: 51.99% vs POE: 0.78%), Shanghai (POI: 58.31% vs POE: 0.69%), Fujian (POI: 14.31% vs POE: 9.81%), Jiangsu (POI: 10.8% vs POE: 5.14%), and Zhejiang (POI: 25.46% vs POE: 7.6%).

**Fig. 6:**
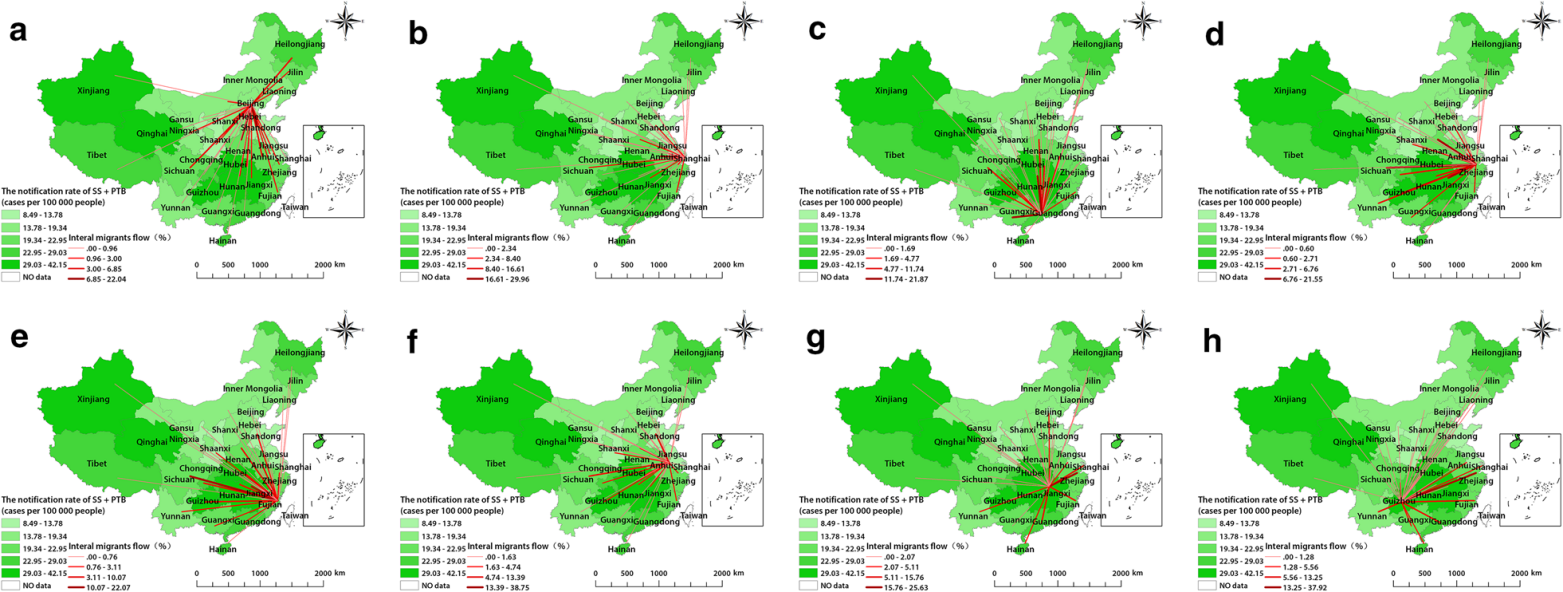
The internal migration flow of Beijing, Shanghai, Guangdong, Zhejiang, Fujian, Jiangsu, Jiangxi, and Guizhou provinces. **a**, **b**, **c**, **d**, **e**, and **f** present the immigrant flow of Beijing, Shanghai, Guangdong, Zhejiang, Fujian, and Jiangsu over 2011–2017, respectively. g and h present the emigrant flow of Jiangxi and Guizhou over 2011–2017, respectively. *TB* tuberculosis, *SS + PTB* sputum smear-positive pulmonary TB

Figure 6 shows the migration flows of internal migrants for the eight spatial clusters. The highest proportion of immigrants from Hebei (22.04%) flowed into Beijing, with immigrants from other spatial clusters accounting for 16.49% of all immigrants. Similarly, the highest proportion of immigrants from Anhui (29.96%) flowed into Shanghai, with the other spatial clusters accounting for 33.58% of immigrants. The highest proportion of immigrants from Anhui (21.55%) flowed into Zhejiang, with the other spatial clusters accounting for 28.92% of immigrants. The highest portion of immigrants from Hunan (21.87%) flowed into Guangdong, with immigrants from the other spatial clusters accounting for 20.57% immigrants. The highest proportion of immigrants from Anhui (38.75%) flowed into Jiangsu, with other spatial clusters accounting for 11.10% of immigrants. The highest proportion of immigrants from Sichuan (20.82%) flowed into Fujian, with other spatial clusters accounting for 31.22% of immigrants. In contrast, 37.91% and 13.25% of the emigrants in Guizhou flowed into Zhejiang and Guangdong, respectively. We also found that 25.63% and 15.04% of the emigrants in Jiangxi flowed into Zhejiang and Guangdong, respectively.

## Discussion

In this study, spatial inequity and spatial variation in temporal trends of SS + PTB in mainland China from 2011 to 2017 were explored using Moran’s *I* statistic and Kulldorff’s scan statistic. The results revealed a decreasing trend in the notification rate of SS + PTB with an average annual decrease in notifications of 9.9%. The high-risk areas of SS + PTB were mainly concentrated in western and southeast China. Further, the results also revealed spatial variation in the distribution of internal migration. Stability of spatial clusters was observed each year during the study period, and the clusters were stable within most provinces.

The global Moran’s statistic results indicated that the spatial inequity in the SS + PTB notification rate became increasingly clustered over time. This finding is consistent with previous research. One potential reason for this finding is the increasing trend and fixed migration patterns for patients to flow from rural areas to prefecture cities or the provincial capital city for better diagnosis and treatment; this could increase the SS + PTB incidence for a specific city and have an impact on the clustering of SS + PTB [9]. However, the Moran’s test can only identify clusters of SS + PTB at a specific time point [33, 34]. Therefore, we used the spatial variation in the temporal trend method to evaluate the spatial variation in temporal trends of SS + PTB based on Kulldorff’s scan statistical methodology.

The spatial variation in temporal trends results showed that the spatial pattern of SS + PTB was changing between 2011–2013 and 2014–2017. Most clusters were located in the south, northeast, and west China. Among these clusters, Guangdong, Fujian, Shanghai, Jiangsu, Guangdong, and Zhejiang are developed and prosperous provinces; internal migrants in these provinces accounted for a large proportion of the population, especially in Guangzhou (the capital city of Guangdong), Hangzhou (the capital city of Zhejiang), and Shanghai. Research in Zhejiang has indicated that nearly one-third of reported TB cases are migrants and the actual rate of TB notification in migrants might be underestimated [35]. Similarly, research in Shanghai showed that increases in internal migration have been associated with increasing rates of TB [36]. However, Beijing was the cluster that exhibited an increasing trend in SS + PTB cases. Aside from the influence of HIV-associated TB and drug-resistant TB, it is believed that internal migration plays an important role in promoting growth in the TB epidemic in Beijing [7, 12]. Guizhou and Jiangxi provinces are also in the southern part of China, near Guangdong, Zhejiang, and Jiangsu provinces. Compared with other provinces in south China, Guizhou and Jiangxi provinces have lower levels of socioeconomic development; a large proportion of emigrants live in poverty with poor medical care [37]. Moreover, we identified three clusters in Xinjiang, Ningxia, and Tibet, in the west of China. Xinjiang and Tibet are the largest political subdivisions in China, accounting for one-fifth of China's total territory. Widespread poverty, disparity of traffic infrastructure, uneven distribution of health infrastructure, and limited knowledge of TB are possible reasons for the high rates of TB in Xinjiang and Tibet [38, 39]. Further, there are also ethnic differences in the prevalence of TB in these autonomous regions; aside from the Han ethnic group, other ethnic groups accounted for 59.9%, 91.83%, and 35.4% of the total population in Xinjiang, Tibet, and Ningxia Autonomous Region, respectively.

The local Moran’s statistic results indicated spatial inequity in the proportions of internal emigrants and immigrants, and the clusters were stable within the study period. The high-high clusters of internal emigrants and low-low clusters of internal immigrants were mainly located in west and south China. In contrast, we found the high-high clusters of internal emigrants were closer to eastern coastal provinces. This may suggest that people in these areas are more likely to emigrate to eastern coastal provinces, or that eastern coastal provinces are more attractive for internal immigrants in these areas.

Previous studies have demonstrated that the number of rural-to-urban migrants has been increasing steadily and associated with increasing rates of TB notification [11, 12, 36]. In the current study, a robust relationship between SS + PTB and internal migration was observed using continuous migration data from 2011 to 2017. The results of the fixed effect and spatial autoregression model suggested that internal emigration was a statistically significant predictor of SS + PTB, while immigration was not a significant predictor. Further, model S1 with the emigration variable was able to explain more variation in SS + PTB than other models. However, the rural-to-urban migration and urban-to-rural migration were not a statistically significant predictor of SS + PTB in models 4 and 5. These results indicate that the emigrant population both in household registration places and residence areas is at increased risk of SS + PTB infection and transmission.

The results of the emigration flow maps indicated that emigrants from Guizhou and Jiangxi provinces likely migrate to Fujian, Zhejiang, and Guangdong provinces. In contrast, the results of the immigration flow maps indicated that immigrants in Beijing, Shanghai, Guangdong, Zhejiang, Fujian, and Jiangsu primarily come from neighbouring provinces. These results are consistent with the spatial clustering distribution of TB cases in China. With the process of reform and opening up, China’s economy has undergone rapid development and the number of internal migrants has increased steadily [40]. By the end of 2017, it was estimated that there were around 7.94, 9.73, 18.52, 7, 12.19, and 17.05 million internal immigrants in Beijing, Shanghai, Guangdong, Zhejiang, Fujian, and Jiangsu, respectively. In general, migration is driven by both push and pull factors. There is a push exerted by poverty in undeveloped regions while, on the other hand, the pull of better economic and social opportunities also encourages people to migrate to developed regions. As shown in Table 2, the main reasons for internal migration were the pursuit of work and business. Moreover, the extent of China’s high-speed railway network makes it easy and affordable for people to migrate to neighbouring provinces within a few hours.

GDP per capita was found to be a statistically significant predictor of SS + PTB in models 1, 2, 3, 4, 5 and 6, indicating that economic development could help to increase the risk of SS + PTB. Poverty is a well-known risk factor for TB [41]. Due to the inequity of economic development and education background, internal migrants who come from rural areas with lower education levels are more likely to engage in physical work [42]. As shown in Table 3, the results indicated that 66.39% of internal migrants had a middle school education or lower and 76.11% of internal migrants had monthly household per capita incomes of less than 7000 CNY (around 1000 US$). It should be noted that while internal migrants are one of the powerful forces driving the economy, on the contrary, economic development also contributes to SS + PTB incidence by internal migration. Moreover, latent TB infection (LTBI) is another critical issue that may play an important role in TB epidemiology in the migrant population [43]. Previous research has indicated that migrants travelling to or from high TB burden regions are at increased risk of acquiring LTBI [11, 44]. While there are many available strategies for diagnosis and treatment of TB, such as the DOTS strategy, the cost-effectiveness of migrant screening for TB remains a consideration [45].

Population density, education level, and the ratio of males to females were statistically significant predictors of SS + PTB in models 2, 3, 5, and 6. On the other hand, urbanisation rate and the number of hospital beds were not statistically significant predictors in models 1–6, respectively. Urbanisation rate was found to be negatively associated with SS + PTB. This means that urban areas that have well-developed public health infrastructure, better-qualified health care workers, and where most of the residents are covered by medical insurance (such as urban resident basic health insurance or urban employee basic health insurance) are less at risk of SS + PTB [46, 47]. As shown in Table 3, in this study, 61.14% of internal migrants had new rural cooperative medical insurance in their household registration. Due to different medical insurance systems, internal migrants who are covered by new rural cooperative medical insurance are required to return their *hukou* registered place [48]. In contrast, the prevalence of TB in rural areas is consistently higher than in urban areas; this highlights a number of issues, such as unbalanced economic development, inequality of basic public health services, and limited healthcare resource allocation [49, 50].

Our study has several limitations that should be noted. First, this study did not include SS + PTB cases of internal migrant. Thus, demographic information for those population was not available. Second, the selection of the optimal scanning window may influence the results of spatial–temporal scan statistics. In this study, due to the latent impact of internal migration, the optimal size of the scanning window was set as the intersection results of 7% to 13%. Further studies should address the influence of migration on the scanning window. Finally, this was an ecological study examining the association between SS + PTB rate and internal migration; the potential ecological fallacy is inevitable.

## Conclusions

In short, this study identified a decreasing trend in the notification rate of SS + PTB from 2011 to 2017. We found spatial inequity of SS + PTB and spatial variation in internal migration in mainland China. The SS + PTB clusters were mainly located in western, southern China and the internal migration clusters were mainly located in central inland China. The proportion of emigrants was negatively correlated with SS + PTB, while the rural-to-urban and urban-to-rural migration were not significantly correlated with SS + PTB. The proportion of emigrants could explain more variation in SS + PTB in the eastern region in mainland China. Further, examination of the characteristics of internal migrants revealed that most had lower education backgrounds and incomes, and most were from rural households. Therefore, we recommend that policymakers acknowledge that migrants are a vulnerable population group for PTB. Cooperative efforts should be strengthened between provinces where there are high proportions of emigrants and immigrants in order to enable effective TB control. Further research is needed to explore the TB epidemic characteristics associated with internal migration based individual migrant data, particularly in high-risk TB areas in China.

## Supplementary information

**Additional file 1: Table S1.** The result of fixed effect and spatial autoregressive model. **Table S2.** Spatial clusters of temporal trends of smear positive PTB in China, 2011–2013. **Table S3.** Spatial clusters of temporal trends of smear positive PTB in China, 2014–2017. **Figure S1.** The annual average notification rate of SS + PTB at the province level in mainland China, 2011-2013 and 2014-2017. **Figure S2.** The spatial variation in temporal trends of smear-positive PTB in China, 2011-2013 and 2014-2017.

## Data Availability

The annually reported all-forms PTB cases from January 2011 to December 2017 in each of 31 provinces of mainland China were obtained from the web-based national Notifiable Infectious Diseases Reporting Information System (NIDRIS). We would like to share statistical results of this study. If anyone needs these data, please contact the corresponding author for a soft copy.

## Abbreviations

TB: Tuberculosis
SS + PTB: Sputum smear-positive pulmonary TB
GIS: Geographical information system
NIDRIS: Notifiable Infectious Diseases Reporting Information System
POE: Proportion of emigrants
POI: Proportion of immigrants
POR: Proportion of rural-to-urban migrants
POU: Proportion of urban-to-rural migrants
GDP: Gross domestic product
PCGDP: Gross domestic product per capita
EDU: College degree or higher
MF: The ratio of male to female
UR: Urbanization rate
PD: Population density
BED: The number of hospital beds
LISA: Local indicator of spatial association
