# Supplementary material for: Spatial inequality, characteristics of internal migration, and pulmonary tuberculosis in China, 2011–2017: a spatial analysis
Source: Infect Dis Poverty. 2020 Nov 19;9:159. doi: 10.1186/s40249-020-00778-0 (PMC7678065; doi:10.1186/s40249-020-00778-0)
Supplement: Supplementary file 1 — Additional file 1: Table S1. The result of fixed effect and spatial autoregressive model. Table S2. Spatial clusters of temporal trends of smear positive PTB in China, 2011–2013. Table S3. Spatial clusters of temporal trends of smear positive PTB in China, 2014–2017. Figure S1. The annual average notification rate of SS + PTB at the province level in mainland China, 2011-2013 and 2014-2017. Figure S2. The spatial variation in temporal trends of smear-positive PTB in China, 2011-2013 and 2014-2017. [file 40249_2020_778_MOESM1_ESM.docx]

**Spatial inequality, characteristics of internal migration, and pulmonary tuberculosis in China, 2011–2017: A spatial analysis**

Wen-Chong He, Master^1,2^, Ke Ju, Master^3,4╀^, Ya-Min Gao, Bachelor^5^, Pei Zhang, Master^6^, Yin-Xia Zhang, PhD^5^, Ye Jiang, PhD^7^, Wei-Bin Liao, PhD^4╀^

^1^ Research management office, West China Second University Hospital, Sichuan University.

^2^ Key Laboratory of Birth Defects and Related Diseases of Women and Children (Sichuan University), Ministry of Education.

^3^ School of Public Health and Preventive Medicine, Monash University, Melbourne, Australia.

^4^ West China School of Public Health and West China Fourth Hospital, Sichuan University, Chengdu, China.

^5^ Department of Health, Northwest Minzu University, Lanzhou, China.

^6^ School of Public Health, The University of Hong Kong, Hong Kong Special Administrative Region.

^7^ School of Geography and Environmental Engineering, Lanzhou City University, Lanzhou, China.

**Running title:** Internal migration and pulmonary tuberculosis in China

**Funding**

This work has not received any funding.

## ^╀^Corresponding to:

Dr. Weibin Liao, West China School of Public Health and West China Fourth Hospital, Sichuan University, Chengdu, No.37 Guoxue Road, Wuhou district, China; Email: [gymlwb@gmail.com](mailto:gymlwb@gmail.com).

Mr. Ke Ju, Department of Epidemiology and Preventive Medicine, School of Public Health and Preventive Medicine, Monash University, Melbourne, Victoria, Australia; Email: [jako1993@163.com](mailto:jako1993@163.com).

**Table S1.** The result of fixed effect and spatial autoregressive model

| Variable |  | Model S1 | Model S2 | Model S3 |  |
| --- | --- | --- | --- | --- | --- |
| lnPOE |  | -0.095(0.047)* | 0.156(0.151) | -0.093(0.127) |  |
| lnPOI |  | 0.09(0.086) | -0.083(0.121) | 0.133(0.172) |  |
| lnPCGDP |  | 0.417(0.146)** | 2.126(0.65)** | 1.217(0.365)** |  |
| lnPD |  | -0.07(0.79) | 0.27(3.498) | 1.391(3.171) |  |
| lnEDU |  | -0.111(0.162) | -0.416(0.155)** | -0.068(0.138) |  |
| lnUR |  | -2.757(0.748)** | -2.605(1.5) | -0.166(0.703) |  |
| lnBED |  | -0.188(0.203) | 0.606(0.35) | -0.503(0.472) |  |
| lnMF |  | -0.528(0.422) | -2.203(0.885)** | -1.519(0.439)** |  |
| Year |  |  |  |  |  |
| 2012 |  | -0.01(0.043)** | -0.409(0.059)*** | -0.282(0.101)** |  |
| 2013 |  | -0.182(0.054)** | -0.668(0.085)*** | -0.528(0.153)** |  |
| 2014 |  | -0.301(0.071)** | -0.928(0.124)*** | -0.793(0.209)** |  |
| 2015 |  | -0.348(0.059)*** | -1.095(0.144)*** | -0.936(0.256)** |  |
| 2016 |  | -0.362(0.07)*** | -1.262(0.175)*** | -1.006(0.283)** |  |
| 2017 |  | -0.374(0.072)*** | -1.449(0.204)*** | -0.977(0.3)** |  |
| Intercept |  | 12.202(4.642)*** | 19.927(18.392) | 3.684(11.866) |  |
| No.Obs |  | 77 | 56 | 84 |  |
| R-squared |  | 0.514 | 0.209 | 0.081 |  |

**Note**: Robust stand-errors are in parentheses. ***, ** and * indicate the significance at 1%, 5%, and 10% level, respectively. POE: Proportion of internal emigrants (%); POI: Proportion of internal immigrants (%); PCGDP: Per capita GDP (10 000 RMB); PD: Population density (1/km^2^); EDU: Proportion of population with college degree or above (%);UR: Urbanization rate (%);BED: The number of hospital beds; MF: the ratio of male to female.

**Table S2.** Spatial clusters of temporal trends of smear positive PTB in China, 2011–2013.

| **Cluster** | **Province** | **Observed cases** | **Expected cases** | **Inside time trend** | **Out time trend** | ***RR*** | ***LLR*** | ***P-*value** |
| --- | --- | --- | --- | --- | --- | --- | --- | --- |
| Most likely cluster | Hunan, Jiangxi | 128805 | 87101 | -4.152 | -14.01 | 1.55 | 442.36 | <0.001 |
| Secondary cluster 1 | Guangdong | 107257 | 82589 | -3.95 | -13.83 | 1.33 | 376.35 | <0.001 |
| Secondary cluster 2 | Xinjiang, Qinghai, Tibet | 44776 | 26252 | -4.3 | -13.23 | 1.74 | 136.90 | <0.001 |
| Secondary cluster 3 | Shanghai, Zhejiang | 43930 | 61327 | -5.33 | -13.17 | 0.7 | 104.49 | <0.001 |
| Secondary cluster 4 | Guizhou | 39090 | 27314 | -5.59 | -13.12 | 1.45 | 86.43 | <0.001 |
| Secondary cluster 5 | Beijing | 6883 | 15846 | +5.01 | -12.96 | 0.43 | 80.03 | <0.001 |
| Secondary cluster 6 | Jiangsu | 35702 | 62040 | -10.61 | -12.95 | 0.56 | 8 | <0.001 |

**Note:** ‘+’ means annual increase trend, ‘-’ means annual decrease trend

**Table S3.** Spatial clusters of temporal trends of smear positive PTB in China, 2014–2017.

| **Cluster** | **Province** | **Observed cases** | **Expected cases** | **Inside time trend** | **Out time trend** | ***RR*** | ***LLR*** | ***P-*value** |
| --- | --- | --- | --- | --- | --- | --- | --- | --- |
| Most likely cluster | Guizhou, Chongqing, Yunnan, Guangxi | 131097 | 115113 | +2.14 | -6.8 | 1.16 | 593.07 | <0.001 |
| Secondary cluster 1 | Shaanxi | 15351 | 27153 | +11.40 | -5.89 | 0.56 | 267.18 | <0.001 |
| Secondary cluster 2 | Jiangxi | 66630 | 32679 | -3.11 | -5.83 | 2.11 | 31.30 | <0.001 |
| Secondary cluster 3 | Xinjiang, Qinghai, Tibet | 44943 | 24254 | -2.91 | -5.74 | 1.89 | 23.36 | <0.001 |
| Secondary cluster 4 | Henan | 56470 | 67908 | -3.37 | -5.78 | 0.82 | 21.18 | <0.001 |
| Secondary cluster 5 | Anhui, Jiangsu | 81713 | 100960 | -4.09 | -5.78 | 0.79 | 14.76 | <0.001 |
| Secondary cluster 6 | Tianjin | 4614 | 10932 | -1.02 | -5.66 | 0.42 | 6.60 | <0.001 |

**Note:** ‘+’ means annual increase trend, ‘-’ means annual decrease trend


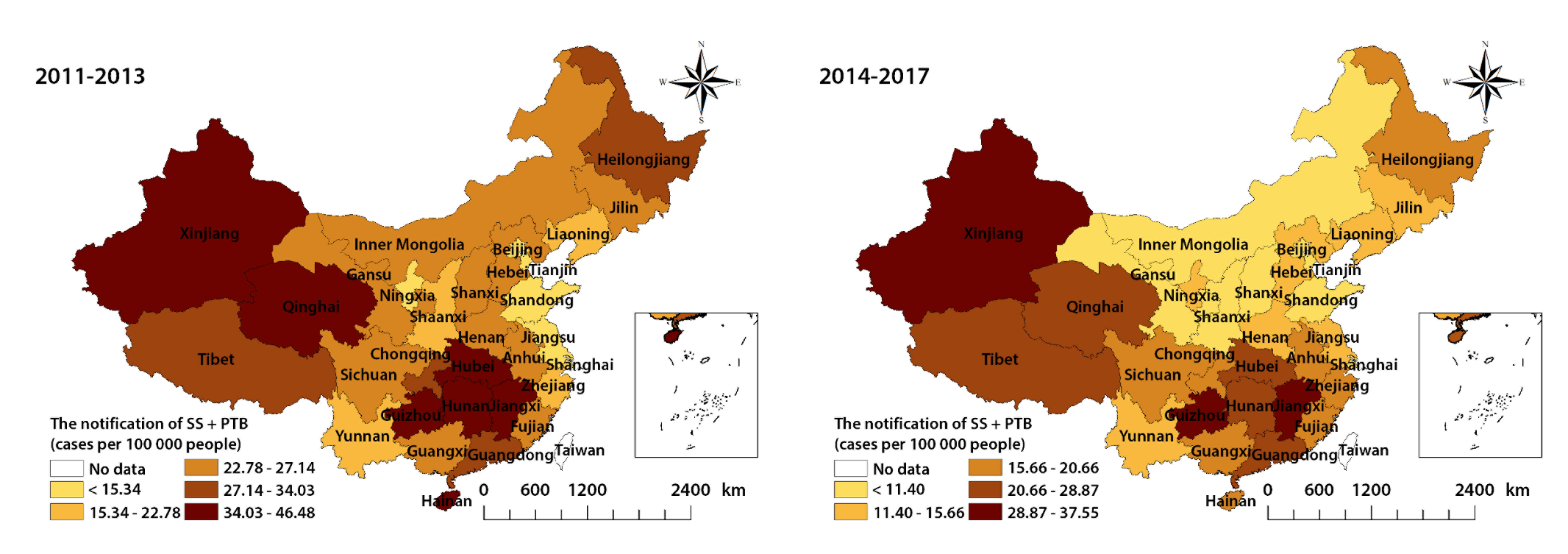


**Figure S1.** The annual average notification rate of SS + PTB at the province level in mainland China, 2011-2013 and 2014-2017.


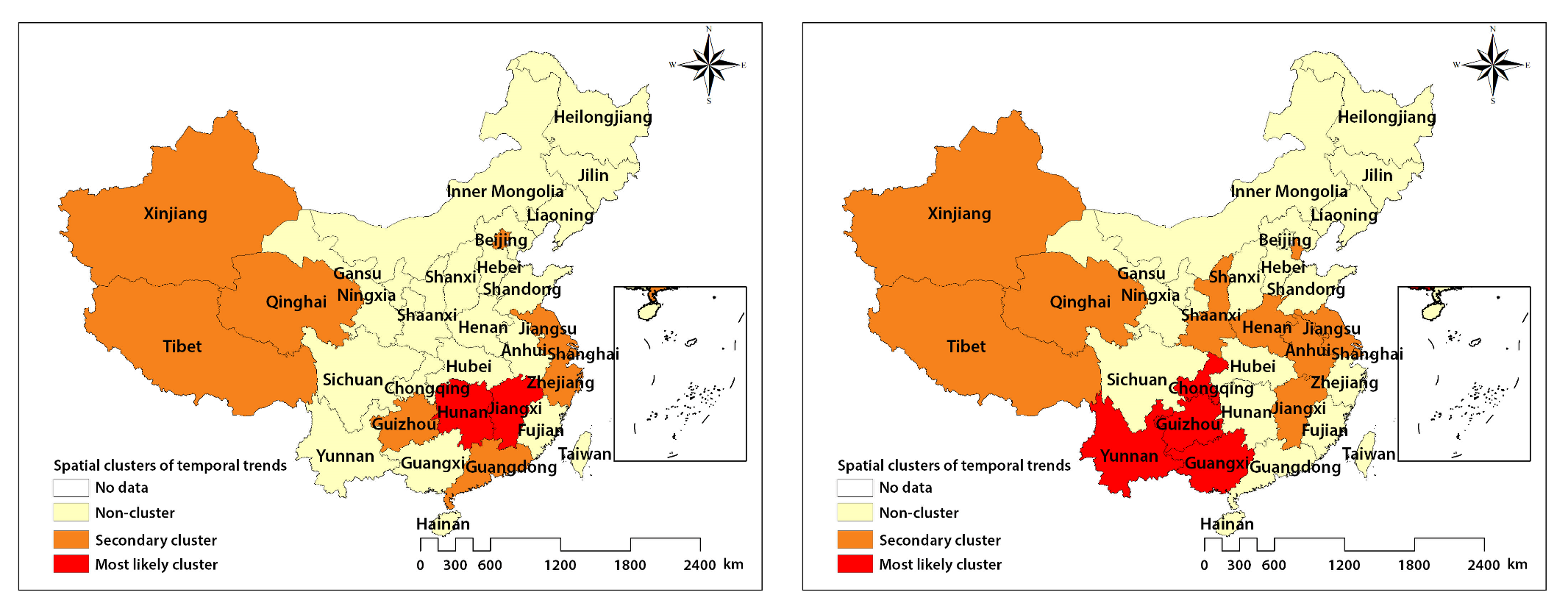


**Figure S2.** The spatial variation in temporal trends of smear-positive PTB in China, 2011-2013 and 2014-2017.
